# Adapting and Testing the Care Partner Hospital Assessment Tool for Use in Dementia Care: Protocol for a 2 Sequential Phase Study

**DOI:** 10.2196/46808

**Published:** 2023-06-22

**Authors:** Beth Fields, Nicole Werner, Manish N Shah, Scott Hetzel, Blair P Golden, Andrea Gilmore-Bykovskyi, Dorothy Farrar Edwards

**Affiliations:** 1 Department of Kinesiology University of Wisconsin-Madison Madison, WI United States; 2 Department of Health and Wellness Design Indiana University School of Public Health-Bloomington Bloomington, IN United States; 3 BerbeeWalsh Department of Emergency Medicine School of Medicine and Public Health University of Wisconsin-Madison Madison, WI United States; 4 Department of Statistics School of Computer, Data and Information Sciences University of Wisconsin-Madison Madison, WI United States; 5 Division of Hospital Medicine, Department of Medicine School of Medicine and Public Health University of Wisconsin-Madison Madison, WI United States; 6 Department of Kinesiology School of Education, University of Wisconsin-Madison Madison, WI United States

**Keywords:** dementia, caregiving, health care, systems, co-design, randomized control trial

## Abstract

**Background:**

Research and policy demonstrate the value of and need for systematically identifying and preparing care partners for their caregiving responsibilities while their family member or friend living with dementia is hospitalized. The Care Partner Hospital Assessment Tool (CHAT) has undergone content and face validation and has been endorsed as appropriate by clinicians to facilitate the timely identification and preparation of care partners of older adult patients during their hospitalization. However, the CHAT has not yet been adapted or prospectively evaluated for use with care partners of hospitalized people living with dementia. Adapting and testing the CHAT via a pilot study will provide the necessary evidence to optimize feasibility and enable future efficacy trials.

**Objective:**

The purpose of this paper is to describe the study protocol for the adaptation and testing of the CHAT for use among care partners of hospitalized people living with dementia to better prepare them for their caregiving responsibilities after hospital discharge.

**Methods:**

Our protocol is based on the National Institutes of Health Stage Model and consists of 2 sequential phases, including formative research and the main trial. In phase 1, we will use a participatory human-centered design process that incorporates people living with dementia and their care partners, health care administrators, and clinicians to adapt the CHAT for dementia care (ie, the Dementia CHAT [D-CHAT]; stage IA). In phase 2, we will partner with a large academic medical system to complete a pilot randomized controlled trial to examine the feasibility and estimate the size of the effect of the D-CHAT on care partners’ preparedness for caregiving (stage IB). We anticipate this study to take approximately 60 months to complete, from study start-up procedures to dissemination. The 2 phases will take place between December 1, 2022, and November 30, 2027.

**Results:**

The study protocol will yield (1) a converged-upon, ready-for-feasibility testing D-CHAT; (2) descriptive and feasibility characteristics of delivering the D-CHAT; and (3) effect size estimates of the D-CHAT on care partner preparedness. We anticipate that the resultant D-CHAT will provide clinicians with guidance on how to identify and better prepare care partners for hospitalized people living with dementia. In turn, care partners will feel equipped to fulfill caregiving roles for their family members or friends living with dementia.

**Conclusions:**

The expected results of this study are to favorably impact hospital-based care processes and outcomes for people living with dementia and their care partners and to elucidate the essential caregiving role that so many care partners of people living with dementia assume.

**Trial Registration:**

ClinicalTrials.gov NCT05592366; https://clinicaltrials.gov/ct2/show/NCT05592366

**International Registered Report Identifier (IRRID):**

PRR1-10.2196/46808

## Introduction

People living with dementia experience frequent and costly hospitalizations, which are associated with poor health outcomes. Compared to other older people, people living with dementia have twice as many hospitalizations and stay nearly a day longer [1]. Moreover, evidence indicates that people living with dementia discharged from hospitals are at higher risk for readmission within 30 days [2,3], urinary tract infections, pressure areas, pneumonia, and delirium [4]. The total costs of these hospital services are more than 3 times greater for people living with dementia than for those without dementia [5]. Further, hospitalizations also hasten the cognitive decline of people living with dementia [6]. Care partners (a traditional family, partner, or other support person designated by the older adult care recipient) have the potential to mitigate these adverse outcomes for people living with dementia when included in care processes during hospitalization.

While more than 11 million care partners of people living with dementia provide nearly 15 billion hours of unpaid care annually [6], many hospitals fail to integrate care partners in the care delivery processes of people living with dementia [7]. Care partners are not included despite our knowledge that frontline approaches to reducing adverse outcomes rely on their unique knowledge of the preferences and functional abilities of people living with dementia [7,8]. In fact, a recent study investigated compliance with the Caregiver Advise, Record, and Enable (CARE) Act, which requires hospitals to (1) provide admitted patients the opportunity to identify and record the name of a care partner; (2) inform the care partner when discharge is to occur; and (3) prepare care partners to fulfill caregiving roles to be performed in the home, like complex medical and nursing tasks [9]. Analyses of more than 200 direct observations and 13,000 patient electronic health records for the first 6 months after implementation of the CARE Act in 1 health care system revealed the following: (1) 53% of patients identified a care partner on admission, 23% declined, and 8% were not asked by a clinician; (2) 97% of care partners identified on hospital admission were not notified of the patient’s upcoming discharge; and (3) only 19% of care partners received preparation on tasks to be performed in the home during the patient’s hospital stay [10,11].

Further, a systematic review of the needs of care partners of people living with dementia across care settings [12] found limited available evidence for identifying and preparing care partners for their caregiving tasks in settings other than the home and community; no studies focused on acute care settings. These findings highlight missed opportunities to individualize care for people living with dementia and to prepare their care partners to fulfill caregiving tasks post hospital discharge. Despite their essential role, care partners who are poorly integrated into hospitalizations and inadequately prepared to fulfill dementia caregiving tasks are at high risk of experiencing excess burden, chronic stress, and depression [13,14].

Clinical decision-support tools offer a valuable resource for addressing this gap in practice as they enable multidisciplinary teams to navigate complex care processes (eg, care partner identification) using structured yet flexible guidance [15]. Common uses of clinical decision-support tools include timely screening for preventable conditions or medical events and ensuring accurate diagnoses [15]. Yet, a recent systematic review uncovered no clinical decision-support tools to guide the systematic identification and preparation of care partners for hospitalized people living with dementia [16].

In response to this gap, we developed and validated a standardized decision-support tool to facilitate the timely identification and preparation of care partners for cognitively unimpaired adult patients during their hospitalization. The Care Partner Hospital Assessment Tool (CHAT) has strong content and face validity and is endorsed as appropriate by clinicians and care partners in the hospital setting [17,18]. Guided by the widely used and effective decision-support model of Screening, Brief Intervention, and Referral to Treatment [19,20], the CHAT applies a sequential screening and referral pathway that (1) identifies care partners and their preferences for involvement in the patients’ hospital care and (2) tailors referrals to address their stated preferences and unmet needs for postdischarge preparedness.

Because there is a great need for a novel decision-support tool in hospital-based dementia care, we will (1) adapt the CHAT for dementia (ie, the Dementia Care Partner Hospital Assessment Tool (D-CHAT)), and then (2) test the feasibility and estimate the size of the effect of the D-CHAT compared to usual care. D-CHAT has the potential to improve hospital-based care processes and outcomes for people living with dementia and their care partners. In addition, findings from this study will further elucidate the essential caregiving role that so many care partners of people living with dementia assume.

## Methods

### Design and Conceptual Framework

The study is based on the National Institutes of Health Stage Model approach [21] to intervention development, focusing on stage 1, and will be conducted in 2 phases: formative research and the main trial. During the formative research phase, we will use a participatory human-centered design process that incorporates people living with dementia and their care partners, health care administrators, and clinicians to refine and adapt the CHAT for hospital-based dementia care (stage IA). During the main trial phase, we will conduct a pilot randomized controlled trial to examine the feasibility and estimate the size of the effect of the D-CHAT on care partners’ preparedness for caregiving tasks (stage IB).

**Figure 1:**
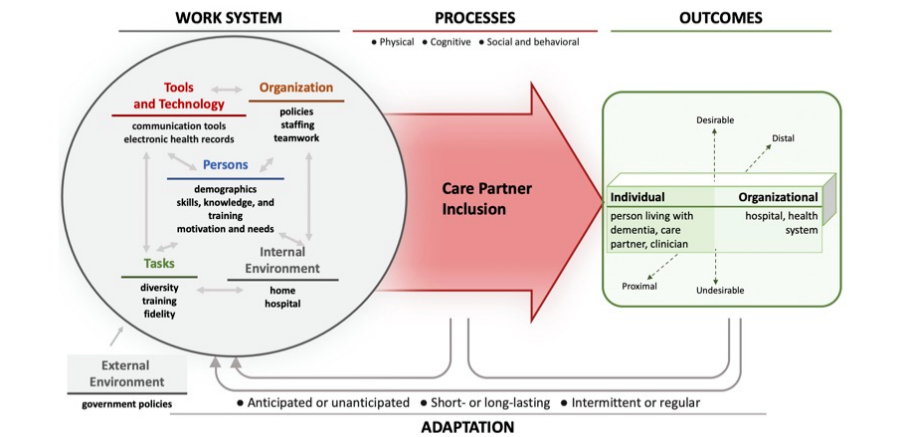
System Engineering Initiative for Patient Safety 2.0 model.

The System Engineering Initiative for Patient Safety 2.0 model (Figure 1) will guide our data collection and analysis to ensure the identification of components influencing the adaptation and delivery of the D-CHAT across all health care system components. This model emphasizes a working system that includes interacting components (ie, people, tasks, tools or technology, organization, and physical environment) that influence health care processes and outcomes [22-24].

### Ethics Approval

The study was approved by the institutional review board on April 11, 2023 (2022-0352). All protocol modifications will be reviewed and approved by the institutional review board as needed.

### Phase 1—Formative Research

#### Sample

Based on established relationships with health system and community partners, we will purposefully select interested parties for two groups: (1) health care administrators and frontline clinicians, and (2) people living with dementia and their care partners that meet eligibility criteria (see Table 1). Each group will form a design team and have 5-7 participants, which is within the standard range used in participatory design research [25]. The small number of participants in groups reduces the potential for groupthink (ie, by having products from one group evaluated by the other) and ensures that all participants have ample time to provide their perspectives [26].

**Table 1:** Sampling plan (N=10-14).

Group			Reason for inclusion		Eligibility criteria
**Group 1 (n=5-7)**					
	Health care administrators	Knowledge and expertise related to the day-to-day operations of hospital, patient eligibility, enrollment, claims, and payment of health services		Have at least 5 years of professional experience in their respective position Speak and understand English	
	Clinicians	Knowledge and expertise related to the delivery of health services		Have at least 5 years of professional experience in their respective position Speak and understand English	
**Group 2 (n=5-7)**					
	People living with dementia	Knowledge and experience related to receiving hospital care		Have experienced a hospitalization Have some form of dementia Identified a care partner	
	Care partners of people living with dementia			Provide unpaid care to a relative or partner living with dementia to help them take care of themselves Be at least 18 years or older Speak and understand English	

#### Data Collection

Teams will complete 5 co-design videoconference sessions occurring in parallel across 4 months, with 2-3 weeks between each session. In session one, we will present the original CHAT. We will lead discussion, interpretation, and respectful debate among design team members to begin to generate the needs of dementia care partners and health care system process changes to optimize their involvement in care. We will ensure progress toward solution convergence from session to session. The design teams will conduct sessions independently, but in between sessions, they will swap solutions and provide feedback, which we will summarize and present in subsequent sessions. Each web-based session will last approximately 90 minutes and will be audio recorded for subsequent analysis. The design process can be conceptualized as a funnel in which we start with divergent ideas and then work toward convergence through the sessions. Participants will receive US $20 after completing each session.

#### Data Analysis

Within the 2-3 weeks between each session, we will review audio recordings as well as any team and participant observation notes, case scenarios, and sketches. Two members of our team will use the Rapid Identification of Themes From Audio Recordings (RITA) method [27] to identify design specifications. The same 2 team members will then combine the themes from the RITA with the synthesized team and participant observation notes, use of case scenarios, and sketches to provide as inputs to participants at the following design session. Following the design session’s completion, we will adapt the CHAT for dementia care, applying input from the final design session, and performing member checking by reviewing changes with members from the groups. This process will produce a converged-upon D-CHAT that is ready for stage 1B feasibility testing [21].

### Phase 2—Main Trial

#### Setting and Sample

This single-blind pilot randomized controlled trial [28] will occur on medical and surgical units at a large academic health care system in the Midwest. This system serves more than 700,000 patients each year with approximately 1849 physicians and 21,000 staff at 7 hospitals. Care partners will be asked to participate if they meet the following inclusion criteria: (1) provide unpaid care to a hospitalized adult relative or partner to help them take care of themselves because of a dementia diagnosis, (2) be at least 18 years or older, and (3) speak and understand English. Care partners of hospitalized adult relatives or partners that are observation or same-day stay patients will be excluded. There will be no exclusion based on sex, gender, race, or ethnicity.

#### Training, Informed Consent, and Screening

**Figure 2:**
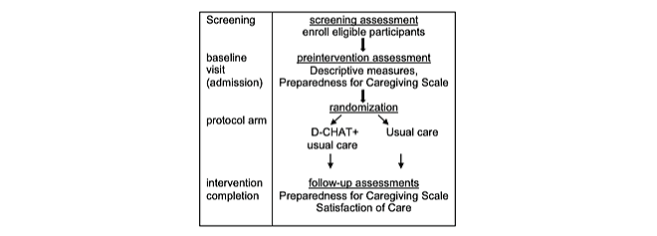
Main trial flow diagram. D-CHAT: Dementia Care Partner Hospital Assessment Tool.

Before recruitment begins, we will provide training on the D-CHAT to clinicians in medical and surgical units. To reduce contamination [29], we will instruct trained clinicians to not use the D-CHAT when treating those in the control arm and to not discuss details of the D-CHAT with nontrained clinicians. Once this training is completed, a member of the study team will work with unit coordinators to schedule morning, afternoon, and evening shifts for data screening, consent, and data collection. Institutional review board approval will be obtained to access electronic health records of hospitalized people living with dementia. We will use electronic health records to identify patients who have a documented care partner (or legally authorized representative). Someone who is part of the patient’s care team will then approach the patient’s present care partners to determine their interest in the study. If a care partner expresses interest in participating, a study team will then share study details, obtain written consent, and complete the baseline visit (see Figure 2). A member of the study team will monitor protocol deviations like unintended crossovers by completing weekly, in-person check-ins with the trained clinicians.

#### Baseline Visit

The baseline visit will be completed in a designated room in the hospital to limit distractions. After completing consent, participants will complete descriptive measures and the primary clinical outcome measure (see Table 2). All measures will be administered by a trained, blind assessor. Immediately after these procedures, participants will be randomized to either the intervention or control group, and the clinical team will be notified of the assignment. These efforts will minimize bias and increase internal validity [28].

**Table 2:** Measures.

Study measures		Instrument	Collection method	Time points	
				1^a^	2^b^
**Clinical outcomes**					
	Preparedness for caregiving	The Preparedness for Caregiving Scale	Self-report	✓	✓
	Satisfaction of care	Hospital Consumer Assessment of Health care Providers and Systems	Self-report		✓
	Burden	Zarit Burden Interview	Self-report	✓	✓
	Depression	Patient Health Questionnaire-2	Self-report	✓	✓
**Descriptive measures**					
	Demographic characteristics	Patient and care partner sex, age, race or ethnicity, education, employment status	Self-report	✓	
	Admission status	Patient reason for patient admission	Medical record	✓	
	Dementia characteristics	Patient type and stage of dementia	Medical record	✓	
	Caregiving context characteristics	Care partner relationship to patient, hours providing care, living situation	Self-report	✓	
**Feasibility measures**					
	Recruitment	Number of care partner contacts, screened, consented, and enrolled	Study records		✓
	Attrition	Reasons for absence and attrition	Study records		✓
	Safety	Adverse events from completing the D-CHAT^c^	Study records		✓
	Time to complete measures and D-CHAT	Minutes	Study records	✓	
	Adherence	Reasons for not following the D-CHAT protocol	Self-report		✓
	Implementation satisfaction	Clinician questionnaire	Self-report		✓

^a^Time point 1: preintervention—within 24 hours of the patient’s admission.

^b^Time point 2: follow-up—within 72 hours of the patient’s discharge.

^c^D-CHAT: Dementia Care Partner Hospital Assessment Tool.

#### Intervention

Care partner participants randomized to the intervention group will receive the D-CHAT and usual care delivered through the patient’s care team. Aftercare partners complete the D-CHAT, and their responses will be given to the unit coordinator to review and make suggested referrals to specified clinicians. These clinicians then use the responses to develop and complete information and skill preparation with the care partners. For example, the D-CHAT could reveal that a care partner does not have the necessary diabetes knowledge or skill set to help give her husband living with Alzheimer disease and diabetes his insulin injections. Information gleaned from the D-CHAT would trigger a diabetes care and education specialist to address the care partners’ caregiving needs.

#### Usual Care Control

The comparison condition will be the usual care that hospitalized people living with dementia and their care partners receive as part of the large academic health care system. No standardized clinical decision-support tool that facilitates the identification and preparation of care partners is currently used for routine hospital-based dementia care. These care partner participants will complete the same measures as participants in the intervention condition on the same timeline using identical assessment procedures.

#### Measures

Participants will be assessed on the measures listed in Table 2. Participants will be compensated US $25 after each time point is completed. Baseline measures will be collected in person at the hospital, and follow-up assessments will be administered via phone by a study team member. The primary clinical outcome we will measure is a change in care partner preparedness. The Preparedness for Caregiving Scale [30] is a self-rated measure that consists of 8 items that ask care partners how well prepared they believe they are for multiple domains of caregiving. Preparedness is defined as perceived readiness for multiple domains of the caregiving role, such as providing physical care, providing emotional support, setting up in-home support services, and dealing with the stress of caregiving. Responses are rated on a 5-point scale, with scores ranging from 0 (not at all prepared) to 4 (very well prepared). The scale is scored by calculating the mean of all items answered with a score range of 0 to 4. The higher the score, the more prepared the care partner feels for caregiving. The items have an internal consistency rating of 0.88 to 0.93. We will also collect secondary outcome measures, including satisfaction of care, caregiving burden, and depression, using standardized measures.

#### Sample Size Justification

An estimated sample size of 128 will be recruited for the main trial based on the 95% CI width around the effect of the D-CHAT on care partner preparedness as measured by the Preparedness for Caregiving Scale [31]. Since this is a novel tool that will be adapted as part of phase 1, the variability of the response is unknown, and therefore justification is based on general terms of SD. With 64 subjects in both groups, the 95% CI width of the estimated effect will be 0.35 × SD. We expect to see a medium effect size (Cohen *d*=0.5). With this 95% CI width and expected effect size, we will have a sufficiently narrow CI of the true effect size to apply toward the planning of the future efficacy trial. If there was a ≈12% loss to follow-up such that 56 in each group have follow-up data, the 95% CI width would only increase to 0.375 × SD.

#### Data Analysis

Analyses will be conducted using SAS (SAS Institute Inc) [32]. We will screen univariate distributions for normality and influential outliers and confirm that the randomization process balanced demographic characteristics between groups. We will also document the number of participants who are lost in each group, the reasons for the loss, and unintended crossovers. This information will influence the design of a future trial, including the frequency and mode of communication with clinicians and participants. The effect of the D-CHAT on care partner preparedness will be estimated as the difference in mean change from baseline to study completion between groups. The 95% CI will be calculated using a *t* distribution. Cohen *d* effect size will be calculated as the effect of the D-CHAT divided by the pooled standard deviation. This will give us a sense of the magnitude of the effect in terms of SDs. If normality assumptions in the raw data are not met and a simple transformation of the data (logarithmic or square root) does not sufficiently normalize the distribution, then we will use a nonparametric equivalent methodology [33]. If greater than 10% of the participants are noncompliant with their group assignment, then a per-protocol sensitivity analysis will be conducted to assess how influential the noncompliance was on the results of the study. Noncompliance is based on whether the group assignment was followed.

## Results

We anticipate this study to take approximately 60 months to complete, from study start-up procedures to dissemination. The 2 phases will take place between December 1, 2022, and November 30, 2027. We expect the following results:

- Phase 1—formative research: Results will provide a converged-upon, ready-for-feasibility testing, D-CHAT, to guide clinicians’ identification and preparation of care partners in hospital care of people living with dementia. The D-CHAT will include caregiving domains unique to dementia caregiving and discipline-specific treatment to meet the needs of care partners.
- Phase 2—main trial: A total of 128 eligible care partners of hospitalized people living with dementia will be randomized into either the D-CHAT plus usual care or usual care-only groups. Descriptive and feasibility characteristics for delivering the D-CHAT will be collected. We expect that care partners who receive the D-CHAT will demonstrate larger improvements in caregiving preparedness than those in the usual care group.

Findings from both studies will be disseminated following best practices throughout the conduct of this research. The study is registered at ClinicalTrials.gov in accordance with current National Institutes of Health (NIH) policies and federal regulations (NCT05592366). Results will be posted to ClinicalTrials.gov within 12 months of study completion. Across all stages of the research, we will publish peer-reviewed papers and present our findings at conferences attended by researchers and clinicians in diverse and relevant fields. We will also share the results of this study directly with relevant and interested parties by developing infographics. These will be distributed through dementia caregiving networks and established partnerships.

## Discussion

Lack of caregiving preparedness is prominent and persistent among care partners of people living with dementia and is associated with increased risk for adverse clinical outcomes for people living with dementia [2,3] and increased levels of burden, depression, and morbidity for care partners. While clinical decision-support tools offer a valuable resource for addressing this gap in practice, no tool exists to guide the systematic identification and preparation of care partners for hospitalized people living with dementia [16].

We will involve a broad range of individuals in the participatory co-design process to create a converged-upon, ready-for-feasibility testing D-CHAT. Moreover, we will use the novel clinical decision-support tool to facilitate the timely identification and preparation of care partners during hospital-based care delivery processes. This work will help bridge the gap between recommendations or policy and practice by providing clinicians with guidance on how to identify and better prepare care partners for hospitalized people living with dementia.

The control group will be randomized to usual care delivered by medical and surgical acute care teams at 1 large academic medical system. While we realize that these teams could prepare care partners for people living with dementia without intervention, we consider the D-CHAT to be more of an individualized care approach that systematically and comprehensively identifies and prepares care partners to fulfill their caregiving responsibilities after the patient discharges from the hospital. The intervention may be seen as burdensome by some clinicians. Our protocol has plans in place to train clinicians on the D-CHAT to ease workflow demands prior to beginning the main trial phase. We are also aware of the potential for measurement bias in the study design (ie, care partners may underreport caregiving needs and preparedness). Therefore, this issue will be discussed during the formative research phase and brought up in the co-design process to develop the best approaches to minimize these potential concerns. We also recognize that hospitals may enforce visiting restrictions at any given point because of the COVID-19 pandemic. For this reason, we have collaborated with research directors and clinical nurse scientists at the academic medical system to identify ways to adapt our data collection and communication approach so that we minimize the potential risks to both hospitalized people living with dementia and their care partners.

Engaging diverse and interested parties to adapt the standardized CHAT for hospital-based dementia care will expand its potential reach. Further, findings from this pilot randomized controlled trial will enable future efficacy trials to advance the refinement and implementation of the decision-support tool that can enhance caregiving preparedness and improve health outcomes after hospital discharge [34]. We anticipate that the resultant D-CHAT will provide clinicians with guidance on how to identify and better prepare care partners for hospitalized people living with dementia. In turn, care partners will feel equipped to fulfill caregiving roles for their family members or friends living with dementia.

## Appendix

Multimedia Appendix 1 Peer-review report by the Career Development for Clinicians/Health Professionals Study Section - National Institute on Aging Initial Review Group (National Institutes of Health, USA).

## Abbreviations

CARE: Caregiver Advise, Record and Enable
CHAT: Care Partner Hospital Assessment Tool
D-CHAT: Dementia Care Partner Hospital Assessment Tool
RITA: Rapid Identification of Themes From Audio Recordings

## Data Availability

The data sets generated during or analyzed during this study will be available from the corresponding author upon reasonable request.

## References

[1] Möllers T, Stocker H, Wei W, Perna L, Brenner H (2019). Length of hospital stay and dementia: a systematic review of observational studies. Int J Geriatr Psychiatry.

[2] Ma C, Bao S, Dull P, Wu B, Yu F (2019). Hospital readmission in persons with dementia: a systematic review. Int J Geriatr Psychiatry.

[3] Phelan EA, Borson S, Grothaus L, Balch S, Larson EB (2012). Association of incident dementia with hospitalizations. JAMA.

[4] Bail K, Berry H, Grealish L, Draper B, Karmel R, Gibson D, Peut A (2013). Potentially preventable complications of urinary tract infections, pressure areas, pneumonia, and delirium in hospitalised dementia patients: retrospective cohort study. BMJ Open.

[5] (2021). 2021 Alzheimer's disease facts and figures. Alzheimer's Association.

[6] James BD, Wilson RS, Capuano AW, Boyle PA, Shah RC, Lamar M, Ely EW, Bennett DA, Schneider JA (2019). Hospitalization, Alzheimer's disease and related neuropathologies, and cognitive decline. Ann Neurol.

[7] Naylor MD, Stephens C, Bowles KH, Bixby MB (2005). Cognitively impaired older adults: from hospital to home: an exploratory study of these patients and their caregivers. Am J Nurs.

[8] Mueller A, Thao L, Condon O, Liebzeit D, Fields B (2021). A systematic review of the needs of dementia caregivers across care settings. Home Health Care Manag Prac.

[9] (2014). State law to help family caregivers. AARP.

[10] Leighton C, Fields B, Rodakowski JL, Feiler C, Hawk M, Bellon JE, James AE (2020). A multisite case study of caregiver advise, record, enable act implementation. Gerontologist.

[11] Fields B, Rodakowski J, Leighton C, Feiler C, Minnier T, James AE (2020). Including and training family caregivers of older adults in hospital care: facilitators and barriers. J Nurs Care Qual.

[12] Fields B, Turner RL, Naidu M, Schulz R, James E, Rodakowski J (2020). Assessments for caregivers of hospitalized older adults. Clin Nurs Res.

[13] The National Academies of Sciences, Engineering, and Medicine (2016). Families Caring for an Aging America.

[14] Bauer M, Fitzgerald L, Koch S, King S (2011). How family carers view hospital discharge planning for the older person with a dementia. Dementia.

[15] Berner ES (2009). Clinical decision support systems: state of the art. Agency for Healthcare Research and Quality.

[16] Butler M, Gaugler JE, Talley KMC, Abdi HI, Desai PJ, Duval S, Forte ML, Nelson VA, Ng W, Ouellette JM, Ratner E, Saha J, Shippee T, Wagner BL, Wilt TJ, Yeshi L (2020). Comparative effectiveness review: care interventions for people living with dementia and their caregivers. Agency for Healthcare Research and Quality.

[17] Fields B, Schulz R, Terhorst L, Carbery M, Rodakowski J (2021). The development and content validation of the care partner hospital assessment tool. Nurs Rep.

[18] Carbery M, Schulz R, Rodakowski J, Terhorst L, Fields B (2021). Evaluating the appropriateness and feasibility of the care partner hospital assessment tool (CHAT). Int J Environ Res Public Health.

[19] Agerwala SM, McCance-Katz EF (2012). Integrating screening, brief intervention, and referral to treatment (SBIRT) into clinical practice settings: a brief review. J Psychoactive Drugs.

[20] (2011). Screening, brief intervention and referral to treatment (SBIRT) in behavioral healthcare. Substance Abuse and Mental Services Administration.

[21] Onken LS, Carroll KM, Shoham V, Cuthbert BN, Riddle M (2014). Reenvisioning clinical science: unifying the discipline to improve the public health. Clin Psychol Sci.

[22] Carayon P, Hundt AS, Karsh BT, Gurses AP, Alvarado CJ, Smith M, Brennan PF (2006). Work system design for patient safety: the SEIPS model. Qual Saf Health Care.

[23] Holden RJ, Carayon P, Gurses AP, Hoonakker P, Hundt AS, Ozok AA, Rivera-Rodriguez AJ (2013). SEIPS 2.0: a human factors framework for studying and improving the work of healthcare professionals and patients. Ergonomics.

[24] Werner NE, Rutkowski R, Graske A, Finta MK, Sellers CR, Seshadri S, Shah MN (2020). Exploring SEIPS 2.0 as a model for analyzing care transitions across work systems. Appl Ergon.

[25] Cheng C, Werner N, Doutcheva Nadia, Warner Gemma, Barton Hanna J, Kelly Michelle M, Ehlenbach Mary L, Wagner Teresa, Finesilver Sara, Katz Barbara J, Nacht Carrie, Coller Ryan J (2020). Codesign and usability testing of a mobile application to support family-delivered enteral tube care. Hosp Pediatr.

[26] Bannon L, Ehn P, Simonsen J, Robertson T (2013). Routledge International Handbook of Participatory Design.

[27] Neal JW, Neal ZP, VanDyke E, Kornbluh M (2015). Expediting the analysis of qualitative data in evaluation. Am J Eval.

[28] Portney LG, Watkins MP (2015). Foundations of Clinical Research: Applications to Practice. 3rd Edition.

[29] Magill N, Knight R, McCrone P, Ismail K, Landau S (2019). A scoping review of the problems and solutions associated with contamination in trials of complex interventions in mental health. BMC Med Res Methodol.

[30] Archbold PG, Stewart BJ, Greenlick MR, Harvath T (1990). Mutuality and preparedness as predictors of caregiver role strain. Res Nurs Health.

[31] Hudson PL, Hayman-White K (2006). Measuring the psychosocial characteristics of family caregivers of palliative care patients: psychometric properties of nine self-report instruments. J Pain Symptom Manage.

[32] (2002). SAS 9.1.3 Documentation. SAS Institute Inc.

[33] Wilcox R (2019). A robust nonparametric measure of effect size based on an analog of Cohen's d, plus inferences about the median of the typical difference. J Mod Appl Stat Methods.

[34] Proctor E, Silmere H, Raghavan R, Hovmand P, Aarons G, Bunger A, Griffey R, Hensley M (2011). Outcomes for implementation research: conceptual distinctions, measurement challenges, and research agenda. Adm Policy Ment Health.

