# Supplementary material for: Adapting and Testing the Care Partner Hospital Assessment Tool for Use in Dementia Care: Protocol for a 2 Sequential Phase Study
Source: JMIR Res Protoc. 2023 Jun 22;12:e46808. doi: 10.2196/46808 (PMC10337334; doi:10.2196/46808)
Supplement: Multimedia Appendix 1 [file resprot_v12i1e46808_app1.pdf]

SUMMARY STATEMENT

(Privileged Communication)

PROGRAM CONTACT:
Melissa Gerald
301-402-4156
geraldmel@nia.nih.gov

Release Date: **06/23/2022**
Revised Date:

Application Number: **1 K23 AG080068-01**

Principal Investigator

FIELDS, BETH

Applicant Organization: UNIVERSITY OF WISCONSIN-MADISON

Review Group: **AGCD-3**
Career Development for Clinicians/Health Professionals Study Section
Career development for clinicians/health care professionals

Meeting Date: **06/06/2022**
Council: **OCT 2022**
Requested Start: **09/01/2022**
RFA/PA: **PA20-206**
PCC: **2BFAMGE**

Project Title: **Adapting and Testing the Care Partner Hospital Assessment Tool (CHAT) for Use in Dementia Care**
SRG Action: **Impact Score:25**
Next Steps: **Visit https://grants.nih.gov/grants/next_steps.htm**
Human Subjects: 30-Human subjects involved - Certified, no SRG concerns
Animal Subjects: 10-No live vertebrate animals involved for competing appl.
Gender: 1A-Both genders, scientifically acceptable
Minority: 1A-Minorities and non-minorities, scientifically acceptable
Age: 3A-No children included, scientifically acceptable

Project Year	Direct Costs Requested	Estimated Total Cost
1	123,280	133,142
2	121,850	131,598
3	119,480	129,038
4	122,865	132,694
5	120,630	130,280
TOTAL	608,105	656,752

ADMINISTRATIVE BUDGET NOTE: The budget shown is the requested budget and has not been adjusted to reflect any recommendations made by reviewers. If an award is planned, the costs will be calculated by Institute grants management staff based on the recommendations outlined below in the COMMITTEE BUDGET RECOMMENDATIONS section.

RESUME AND SUMMARY OF DISCUSSION: This application for a Mentored Patient-Oriented Research Career Development Award (Parent K23, Independent Clinical Trial Required) submitted by The University of Wisconsin at Madison (UWM), WI, on behalf of Dr. Beth Fields, requests five years of support to transition the candidate to an independent clinical research career focusing on the development and testing of tools for Dementia Care. The candidate is an occupational therapist (OT) and a tenure-track Assistant Professor at UWM. Dr. Fields has a PhD in OT and completed a post-doctoral fellowship in Health Policy and Caregiving Research and Health Services Research. The applicant is also affiliated with the School of Nursing (SoN) at UWM the Wisconsin Alzheimer's Disease Research Center (ADRC) and is an executive committee member of the Wisconsin Institute for Healthcare Systems Engineering. Dr. Fields has excellent track records of publications (28 total, 16 as first or senior author) and independent pilot funding from foundations; he is principal investigator (PI) on an NIH R03 and is the recipient of an NIH loan repayment grant award and other professional, even international, career awards. The career development plan (CDP) is designed to pursue the applicant's long-term goal of improving hospital-based care of patients with Alzheimer's disease (AD) and AD-related dementias (ADRD) with focus on unpaid caregivers. Five training goals are identified by the analysis of the training gaps, including participatory human-centered design principles, (2) hospital-based ADRD care delivery, (3) clinical trial design and statistical analysis, (4) ethical and regulatory standards in ADRD research, and (5) professional skills. The CDP incudes an effective plan to achieve the training goals based on hands-on mentored activities, didactics, coursework, meeting attendance, and concrete plans for an R-level grant submission. The research plan (RP) focuses on the preparation of unpaid caregivers to provide home care to AD/ADRD patients; it proposes to adapt the Care Partner Hospital Assessment tool (CHAT) to AD/ADRD (CHAT-AD), test effectiveness and acceptability of the adapted tool, and conduct a pilot randomized clinical trial (RCT) to test effectiveness of CHAT-AD. The proposed approach is supported by preliminary data from the CHAT earlier development; properly powered, well conceived, and tackle an important aspect of AD/ADRD care. The mentorship team is excellent, highly qualified, and committed to the applicant. There is track record of collaborations already in place between the applicant and the mentoring team. The team includes the primary mentor, Dr. Farrar-Edwards (UWM; expert on hospital care and AD/ADRD); three co-mentors, including Drs. Werner (UWM; expert on technology and dementia care), Shah (UWM; expert in acute care for older individuals with AD/ADRD), and Hetzel (UWM; expert clinical biostatistician); two collaborators, Drs Schulz (University of Pittsburgh; expert in psychiatry and geriatrics) and Gilmore-Bykoskyi (UWM); a few consultants with complementary roles complete the mentorship team. There is strong unconditional-to-the-award, institutional support, including 75%-protected research time for research and training, support for the primary mentor's time, and start-up funds. The environment at UWM will effectively support the candidate. Weaknesses include the lack of substantive AD/ADRD clinical and didactic training; the RP does not consider the potential bias arising from the fact that the CHAT+standard care (intervention) group, and the standard care (control) group are handled by the same operators; Aims 1 and 2 are interdependent; different stages of AD/ADRD are conflated in one big group, which will confound results; the pilot RCT is very small and might not provide enough preliminary data to support the larger RCT planned for the R01 submission; and the mentorship team is large and the management of its input will be laborious. In conclusion, the enthusiasm elicited by the application's strengths is somewhat reduced by the training and RP weaknesses.

TRAINING IN THE RESPONSIBLE CONDUCT OF RESEARCH: Acceptable. The planned activities satisfy the requirement for training in the responsible conduct of research.

DESCRIPTION (provided by applicant): This K23 application proposed a career development plan to help Dr. Beth Fields establish an independent research program that focuses on optimizing hospital-based care and outcomes for patients living with Alzheimer's disease and related dementias (ADRD) and their family member or friend care partners. She will train under the mentorship of a transdisciplinary group of senior scientists with research expertise in participatory human-centered design, conduct of clinical trials, and clinical ADRD care. She will continue working with her current

mentors, Drs. Farrar-Edwards, Werner, and Shah, and Mr. Hetzel, all of whom have experience mentoring trainees. This will be complemented by content and mentoring expertise from Drs. Schulz and Gilmore-Bykovskyi. Collectively, this team will provide an outstanding training environment that will allow Dr. Fields to fill critical gaps in her knowledge and skill set relating to the study of hospital-based care processes and outcomes for patients living with ADRD and their care partners. Her training goals are to develop skills in (1) participatory human-centered design principles, (2) hospital-based ADRD care delivery, (3) clinical trial design and statistical analysis, (4) ethical and regulatory standards in ADRD research, and (5) professional skills in team science and scientific leadership. Achieving these goals will strengthen her scholarly activities, establish important collaborations, and acquire critical data that will ensure her successful transition to independence. To this end, Dr. Fields' proposed research plan builds directly on her prior work developing and validating the Care Partner Hospital Assessment Tool (CHAT). Guided by the widely used and effective decision-support model of Screening, Brief Intervention and Referral to Treatment (SBIRT), the CHAT applies a sequential screening and referral pathway that 1) identifies care partners and their preferences for inclusion in the patients' hospital care and 2) tailors referrals to address their stated preferences and unmet needs for post-discharge preparedness. The SBIRT model was designed to adapt flexibly to different health conditions and contexts, thus enabling the adaptation of the CHAT to facilitate the inclusion and preparation of care partners of patients living with ADRD. Therefore, the purpose of this proposal is to adapt CHAT for care partners of hospitalized patients living with ADRD (CHAT-AD) and evaluate its feasibility and potential efficacy in a pilot randomized clinical trial. Findings from this study, in combination with the career development plan, will enable Dr. Fields to launch an independent program of research that aims to (1) improve hospital-based care processes and outcomes for patients living with ADRD and their care partners, and (2) elucidate the essential caregiving role that so many care partners of patients living with ADRD assume.

PUBLIC HEALTH RELEVANCE: Lack of caregiving preparedness is prominent and persistent among care partners of patients living with Alzheimer's disease and related dementias (ADRD) and associated with increased risk for adverse clinical outcomes for patients living with ADRD, and increased levels of burden, depression, and morbidity for care partners. Engaging key stakeholders to adapt the standardized Care Partner Hospital Assessment Tool for hospital-based dementia care will expand its potential reach. Further, findings from this pilot randomized controlled trial will enable future efficacy trials to advance the refinement and implementation of the decision- support tool that can enhance caregiving preparedness to improve health outcomes after hospital discharge.

DISCLAIMER: Please note that the following critiques were prepared by the reviewers prior to the Study Section meeting and are provided in an essentially unedited form. Although there is opportunity for the reviewers to update or revise their written evaluation, based upon the group's discussion, there is no guarantee that individual critiques have been updated after the discussion at the meeting. Therefore, the critiques may not fully reflect the final opinions of the individual reviewers at the close of group discussion or the final majority opinion of the group. Thus, the Resume and Summary of Discussion is the final word on what the reviewers considered critical at the meeting.

CRITIQUE 1:

Candidate: 1
Career Development Plan/Career Goals/Plan to Provide Mentoring: 1
Research Plan: 3
Mentor(s), Co-Mentor(s), Consultant(s), Collaborator(s): 1
Environment Commitment to the Candidate: 1

Overall Impact:

This is a new K23 application, submitted by Dr. Fields, Assistant Professor in the Occupational Therapy Program, Department of Kinesiology at UWM. The project is entitled, “Adapting and Testing the Care Partner Hospital Assessment Tool (CHAT) for Use in Dementia Care”. The candidate has a PhD in Occupational Therapy and has completed a post-doctoral fellowships in Health Policy and Caregiving Research and Health Services Research. She is also an affiliate faculty member in the School of Nursing and Wisconsin Alzheimer’s Disease Research Center (ADRC) and executive committee member of the Wisconsin Institute for Healthcare Systems Engineering. Briefly, the proposed research builds upon the candidate’s prior work developing and validating the Care Partner Hospital Assessment Tool (CHAT), which applies a screening and referral pathway that 1) identifies care partners and their preferences for inclusion in the patients’ hospital care and 2) tailors referrals to address their stated preferences and unmet needs for post-discharge preparedness. The work proposed adapts CHAT for care partners of hospitalized patients living with ADRD (CHAT-AD) and evaluates feasibility and potential efficacy in a pilot randomized clinical trial (RCT). The goal is to apply for R01 funding for a full scale RCT of the CHAT-AD. Her training goals are to develop skills in (1) participatory human-centered design principles, (2) hospital-based ADRD care delivery, (3) clinical trial design and statistical analysis, (4) ethical and regulatory standards in ADRD research, and (5) professional skills in team science and scientific leadership. The application includes an appropriate training plan to accomplish these goals. She is supported by an excellent, highly qualified and committed team of mentors and advisors. Excellent institutional environment and support for the candidate. Overall, there is high enthusiasm about the Candidate; the application includes a few minor concerns related to the RP that are likely addressable.

1. Candidate:
Strengths
- Candidate has PhD in Occupational Therapy, Board Certified in Gerontology by the American Occupational Therapy Association (1 of only 79 in US).
- Complete post-doctoral fellowships in Health Policy and Caregiving Research and Health Services Research.
- Recruited to UWM 2019, tenure-track position.
- Well connected with resources and faculty locally as she is an affiliate faculty member in the School of Nursing, the Division of Geriatrics and Gerontology, and Wisconsin Alzheimer’s Disease Research Center and executive committee member of the Wisconsin Institute for Healthcare Systems Engineering.
- 28 peer reviewed papers (12 first author; 4 senior author).
- Prior funding for research, including Carl and Caroline Swanson Foundation Fellowship through Colorado State University; fellowship support from the Pittsburgh Foundation (UN2016-85266; PI James); 6 pilot grant awards; and as PI on NIA R03 Small Research Grant project (R03AG062848) that developed and validated the Care Partner Hospital Assessment Tool (CHAT).
- NIA Loan repayment award recipient.
- Research awards from American OT Foundation and Canadian Association of Occupational Therapists.

Weaknesses
- None noted.

2. Career Development Plan/Career Goals and Objectives:
Strengths
- Overarching career goal is to establish an independent and NIH-funded research program focused on improving hospital-based care processes and outcomes for patients living with ADRD and their care partners.
- Strong educational and training background, yet clearly justifies additional training need to address identified gaps: including experience conducting studies that incorporate participatory

human-centered design principles, leading randomized clinical trials, and working directly with hospitalized patients living with ADRD and their care partners.
- Training plan includes appropriate coursework, including Advanced Dementia Care certificate course, Clinical Trails, and others; Directed readings with specified mentors; 300hr clinical observation; NIH RCT summer institute; K-to-R seminar at UW; and involvement in national scientific organizations. Each component has indicated deliverable.
- Clinical shadowing with Dr. Golden (hospitalist, dementia expertise).
- Plans for multi-site R01 level study to determine the efficacy of a standardized decision-support tool on caregiving preparedness of care partners and service utilization of patients living with ADRD. The R01 will be designed and powered to detect differences in adverse health and service utilization outcomes, such as urinary tract infections and 30-day hospital readmissions.

Weaknesses
- Unclear if intent is to attend and present at all 4 national organization meetings every year, which would likely be too much.
- The amount of effort focused on career development, research, other clinical or teaching duties is not made explicit in the application.

3. Research Plan:
Strengths
- Extremely important area of research; compelling background evidence and policy support for intervention development in this area.
- Preliminary work, support by R03, developed and validated a standardized decision-support tool to facilitate the timely inclusion and preparation of care partners of cognitively unimpaired adult patients during their hospitalization. The Care Partner Hospital Assessment Tool (CHAT) was found to have strong content validity and is endorsed as feasible and appropriate by clinicians and care partners in the hospital setting.
- The CHAT design follows the clinical decision-support model of screening, Brief Intervention and Referral to Treatment (SBIRT), and was built upon an assessment framework recommended by the National Center on Caregiving at the Family Caregiving Alliance. The tool uses 22 items all of which have a content validity index at or above the acceptable 78% cut point and a scale-content validity index of 85%, based upon surveys with experts in gerontology, caregiving, and health services. Feasibility of the CHAT was also supported by qualitative assessment with health care administrators, clinicians, and care partners.
- The goal of this application is to adapt the CHAT for care partners of patients with ADRD.
- The work proposed involves 2 Aims and is guided by the System Engineering Initiative for Patient Safety (SEIPS) 2.0 conceptual model.
- Aim 1 Employs an iterative human-centered design process to adapt CHAT for care partners of hospitalized patients living with ADRD (CHAT-AD). By forming two stakeholder design teams, one team comprised of previously hospitalized patients with ADRD and their care partners (N=7 dyads) and the second healthcare system administrators and clinicians (N=7). Each will complete 5 co-design videoconferences, 90 minutes, audio recorded for transcription.
- Through iterative steps, the CHAT will be adapted becoming the CHAT-AD, which will include caregiving domains unique to ADRD care partners and discipline-specific.
- Treatment to meet the preparation needs of care partners.
- Aim 2 will conduct a pilot RCT to evaluate the feasibility and estimate the size of the effect of the CHAT-AD compared to usual care. Primary outcome caregiving preparedness and secondary outcome satisfaction with care will be measured pre and post in comparison to the control group. Feasibility measures will include recruitment, attrition, safety, adherence, and implementation satisfaction.
- Partnering with the Acute Care for Elders (ACE) program the study will recruit over 2.5 years 128 eligible care partners of hospitalized patients living with ADRD, who will be randomized into either the CHAT-AD plus usual care or usual care-only groups.

- Sample size estimate appears to provide adequate power.

Weaknesses

- Very little race/ethnic diversity in the study sample. While the sample is local and thus feasible, it is strongly recommended increasing efforts to add diversity, especially in preparation for the R01 application.
- While the ACE team is advised not to use the CHAT-AD with control group patients, bias may be created by training. Would consider the possibility of baseline/control measurement period prior to training and starting the intervention, or other ways to minimize this bias. This is also an important design issue to consider and prepare for considering the R01 submission.
- The team may want to consider enrollment of eligible patients not cared for by the ACE team model as usual care. While achieving an effect in the setting of ACE care would be a robust finding, ACE team care is not available at many hospitals and the effect may be larger in settings without it.
- Caregiving context characteristics are only measured at Time 1 and could change significantly post-discharge at Time 2.
- The planned R01 will be designed and powered to detect differences in adverse health and service utilization outcomes, such as urinary tract infections and 30-day hospital readmissions but these outcomes are not being assessed in Aim 2. Could data from the electronic health record (HER) be leveraged for longer term follow-up?
- A component of the background justification is that care partner who are in adequately prepared are at high risk of experiencing excess burden, chronic stress, and depression. The team could consider exploratory/secondary measures of these constructs as well.

4. Mentor(s), Co-Mentor(s), Consultant(s), Collaborator(s):

Strengths

- Excellent mentoring team with established relationship with the candidate and with each other.
- Strong and personalized letters of support from mentors.
- Primary mentor: Dr. Farrar-Edwards, PhD, is Vilas Distinguished Achievement Professor of Kinesiology and Medicine, Associate Dean of Research for the School of Education, Director of the Collaborative Center for Health Equity, and Outreach, Recruitment and Engagement Lead in the Wisconsin ADRC at the UW.
- Dr. Farrar-Edwards has an outstanding track record of funding, expertise in clinical trials in the hospital setting and content areas including ADRD and caregiving. She has extensive experience mentoring K awardees.
- Co-mentor: Dr. Werner, PhD Associate Professor in the College of Engineering, affiliate faculty of the Division of Geriatrics in the School of Medicine and Public Health, and Director of WIHSE at UWM. She is on the leadership team of the ADRC Care Research Core as the technology and dementia care expert. Will provide support for Aim 1.
- Co-mentor: Dr. Shah, MD Professor of Emergency Medicine, Geriatrics, and Population Health, Vice Chair of Research in the Department of Emergency Medicine, and Director of the ICTR KL2 Program. His clinical and research expertise is acute care for older adults, specifically those with ADRD, excellent track record for funding and mentoring early career investigators. K24 focused on ADRD.
- Co-mentor: Dr. Hetzel, MS, Biostatistics with expertise in clinical trials design and evaluation. Will support Aim 2.
- Dr. Schulz, PhD is a collaborator and past mentor, he is Distinguished Service Professor of Psychiatry, Director of Gerontology and Center for Caregiving Research, Education, and Policy at the University of Pittsburgh.
- Andrea Gilmore-Bykoskyi, PhD is an Associate Professor at the UWM School of Nursing, Deputy Director of the Center for Health Disparities Research, and Informatics; Lead in the Wisconsin ADRC Care Research Core. She has excellent track record of mentoring and will provide support for recruitment strategies and ethical and regulatory issues for ADRD research.

- Additional consultants include Drs. Farsetta (community outreach specialist), Arsenault (nursing, recruitment support), Chapman (director of ACE team), Hirvela (Geriatrics nurse specialist), Golden (hospitalist), and Mr. Zerrenner (care partner to wife and Board Vice Chair for the Fox Valley Memory Study.

Weaknesses

- Big team of mentors, collaborators and consultants will require careful management to be used effectively and efficiently. Some details of meetings with and managing input from this group are limited.

5. Environment:
Strengths

- Strong letter of support from the Department Chair, assuring 75% time for the research and training proposed, support for her primary mentor’s time, and start-up funds to support her research.
- Start-up funds to establish candidate’s Geriatric Health Services Research Laboratory, which support hiring a lab coordinator, graduate research assistant and student who will be assisting with subject recruitment, enrollment, and data management for this application.
- Support from variety of local resources, including the ADRC’s Care Research Core; Institute for Clinical and Translational Research (CTSI); the Wisconsin Institute for Healthcare Systems Engineering; the Wisconsin-Madison Center for Health Disparities Research; the Center for Aging Research and Education in the School of Nursing. The connection to many of these resources is assured by her mentors.
- Letters attesting to support, collaboration, and resources from all involved entities.

Weaknesses

- None noted.

Study Timeline:
Strengths

- Provided and appropriate.

Weaknesses

- None noted.

Protections for Human Subjects:
Acceptable Risks and Adequate Protections.

Data and Safety Monitoring Plan:
Acceptable.

Inclusion Plans:

- Sex/Gender: Distribution justified scientifically.
- Race/Ethnicity: Distribution not justified scientifically. Very little race/ethnic diversity in the study sample.
- Inclusion/Exclusion Based on Age: Distribution justified scientifically.

Training in the Responsible Conduct of Research:
Acceptable.

Resource Sharing Plans:
Acceptable.

Budget and Period of Support:
Recommend as Requested.

CRITIQUE 2:

Candidate: 1
Career Development Plan/Career Goals/Plan to Provide Mentoring: 1
Research Plan: 4
Mentor(s), Co-Mentor(s), Consultant(s), Collaborator(s): 1
Environment Commitment to the Candidate: 1

Overall Impact:
Dr. Fields holds a PhD in OT and is a tenure-track Assistant Professor in the Department of Kinesiology. She has intramural and extramural grants, and over 28 publications with 12 first author and 4 senior authors. Her career training goals are appropriate for K23 award. Her focus is on caregivers of patients with ADRD. She has 2 Aims in her RPs. One to develop CHAT-AD, and to conduct a pilot study using CHAT-AD to prepare unpaid caregivers of ADRD patients for home care. The mentors and environment are outstanding and meets her needs.

1. Candidate:
Strengths
- Dr. Fields earned MS and PhD in OT and Rehabilitation Science from Colorado State University, Postdoctoral Fellow in Health Policy and Caregiving from the University of Pittsburgh, and Health Services Research from the Veteran Administration in Pittsburgh. She is an Assistant Professor in tenure-track in the OT program, Department of Kinesiology, Affiliate faculty member in the SoN and Wisconsin ADRC, executive committee member of the Wisconsin Institute for Healthcare Systems Engineering at the UWM.
- She has 28 peer reviewed paper with 12 first author and 4 senior author.
- She has 6 intramural pilot grants, a foundation grant, National Institute on Disability, Independent Living, and Rehabilitation Research (NIDILRR) grant, and NIA R03 on aging and caregiving research.
- She has existing relationships with her mentor, co-mentors, collaborators, and consultants.

Weaknesses
- None noted.

2. Career Development Plan/Career Goals and Objectives:
Strengths
- She already has knowledge and skill set in caregiving, gerontology, methods of evaluating health services delivery, and clinical decision-support tool development. She Aims to develop geriatric health services with focuses on ADRD caregiving**.**
- Funding for 5 years at 75% time.
- The training Plan via coursework/self-study/seminars involve areas of 1)Participatory human-centered design, 2) hospital-based ADRD care, 3) clinical trial design and statistical analysis, 4) ethical and regulatory issues, especially for ADRD research, 5) leadership, management, presentation, and mentoring skills.

Weaknesses
- None noted.

3. Research Plan:
Strengths
- The goals include development of decision-support tools to facilitate the inclusion and preparation of unpaid family member or friend care partners in hospital-based care; and

examination of decision-support tools and nonpharmacological interventions designed to enhance quality of care and outcomes of adults living with ADRD and their care partners.
- Specifically, the objectives are to adapt the validated CHAT for use in dementia care and determine the impact of the adapted tool (CHAT-AD) on clinical outcomes in care partners of hospitalized patients living with ADRD.
- Aim 1: Adapt CHAT for care partners of hospitalized patients living with ADRD. Team 1 discharged ADRD patients and their care partners (7 dyads), team 2 healthcare system administrators and clinicians (n=7). Each team will co-design videoconference sessions that occur in parallel across 4 months, with 2-3 weeks between each session. Create a usable CHAT-AD prototype that facilitates the timely inclusion and preparation of care partners of hospitalized patients living with ADRD.
- Aim 2: Use CHAT-AD n=64 control, n=64 using CHAT-AD to prepare for post-hospitalization ADRD patient care and referrals as needed to provide care.

Weaknesses
- Patient's AD stage has not been considered in Aims 1 or 2.
- It is not clear how long the patient has carried the ADRD diagnosis and whether caregiver has accepted the diagnosis and their roles.
- Aim 2 depends on the success of Aim 1.
- The type of clinicians and administrators were not detailed.

4. Mentor(s), Co-Mentor(s), Consultant(s), Collaborator(s):
Strengths
- Dr. Farrar-Edwards, PhD Psychology-Aging and Development. Associate Dean for Research, Professor in the Department of Kinesiology, and Medicine. Role= primary mentor. She has funding, publication record, and mentoring experience.
- Dr. Werner, BS, MS, PhD Psychology –Human Factors and Applied Cognition, Associate Professor with Tenure Industrial and Systems Engineering. Director of the Wisconsin Institute of Health Systems Engineering and co-Leader of the UW ICTR Learning Health Systems initiative. She will serve as co-mentor. She has funding, publication record and mentoring experience.
- Dr. Shah, MD, MPH, Professor of Emergency Medicine, Population Health Sciences, and Geriatrics. He will serve as co-mentor. He has funding, publication record, and mentoring experience.
- Dr. Hetzel, MS on Biostatistics. He will serve as co-mentor for biostatistics.
- Collaborators include Drs. Schulz, PhD on caregiving and aging will guide on career development activities, and Gilmore-Bykovskyi, PhD, will mentor in recruitment strategies, ethical, and regulatory issues on ADRD research.
- Consultants include Drs. Farsetta (support recruitment), Zerrenner (insight in ADRD care and caregiving and support recruitment), Arsenault-Knudsen (support recruitment), Chapman (support recruitment and conduct of the proposed study Aim 2), Hirvela (will be observed in her clinical activities by the applicant), and Dr. Golden (will be shadowed in inpatient care setting by the applicant).

Weaknesses
- None noted.

5. Environment:
Strengths
- Aging-Related Resources include: 1) Wisconsin ADRC, funded by NIH-NIA; 2) UWM-Department of Medicine, Division of Geriatrics and Gerontology has in excess of $60 million in funding; 3) Institute on Aging; 4) Wisconsin Alzheimer's Institute; and 5) Center for Aging Research and Education.

- Institutional Resources include: 1) UWM, 2) UWM SOMPH 3) UWM-Dept. of Kinesiology 4) Geriatric health Services Research Laboratory, 5) Wisconsin Institute for Health Systems Engineering, 6) CTSI , 7) UWM Health, 8) UWM-Women's Faculty Mentoring Program.

Weaknesses
- None noted.

Protections for Human Subjects:
Acceptable Risks and Adequate Protections.

Inclusion Plans:
- Sex/Gender: Distribution justified scientifically.
- Race/Ethnicity: Distribution justified scientifically.
- Inclusion/Exclusion Based on Age: Distribution justified scientifically.

Training in the Responsible Conduct of Research:
Acceptable.
Format:
- Format provided.

Subject Matter:
- Subject matter provided.

Faculty Participation:
- Faculty participation provided.

Duration:
- Duration provided.

Frequency:
- Frequency provided.

Resource Sharing Plans:
Acceptable.

Budget and Period of Support:
Recommend as Requested.

CRITIQUE 3:

Candidate: 1
Career Development Plan/Career Goals/Plan to Provide Mentoring: 3
Research Plan: 4
Mentor(s), Co-Mentor(s), Consultant(s), Collaborator(s): 2
Environment Commitment to the Candidate: 1

Overall Impact:
Dr. Fields is an Assistant Professor in the OT Program, Department of Kinesiology at the UWM. She is highly productive and accomplished, and now wishes to apply her prior work focused on caregivers to individuals with dementia and their caregivers. The CDP is well laid out but lacks clinical exposure and national networking experiences in the dementia research field. The RP as laid out also needs to be strengthened in terms of more specificity of the individuals with dementia and their caregivers as well as appropriate study setting.

1. Candidate:
Strengths

- Dr. Fields is an Assistant Professor in the OT Program, Department of Kinesiology at the UWM.
- The candidate has a significant number of publications in the relevant area of caregiving.
- The candidate has been very successful in receiving institutional pilot grants, foundation grants and NIH R03.
- The candidate has been recognized for her leadership in different national societies.

Weaknesses

- None noted.

2. Career Development Plan/Career Goals and Objectives:

Strengths

- Overall goal stated as establishing an independent and NIH funded research program focused on improving care process and outcomes for patients living with AD/ADRD and their care partners.
- Proposing a CDP to strengthen skills in participatory human-centered design processes.
- Development of decision-support tools to facilitate the inclusion and preparation of unpaid family member or friend care partners in hospital-based care.
- Examination of decision-support tools and nonpharmacological interventions designed to enhance quality of care and outcomes of adults living with AD/ADRD) and their care partners.

Weaknesses

- For transitioning to ADRD field, there is no specific clinical observation experience with experts in the dementia field; clinical observation is mentioned in a very generic manner.
- In patient care setting is unlikely to provide insight into caregivers for patients with dementia as visitations are greatly limited in the hospitals at this time. It is unclear what type of clinical exposure Dr. Hirvela will provide.
- In terms of national meetings or training network, the only meeting that is mentioned is Alzheimer’s Association International Conference (AAIC).

3. Research Plan:

Strengths

- Using CHAT, which the candidate has validated and has significant experience.
- Focusing on caregivers and understanding of the role that they play in patient’s hospitalization and its after care is important.

Weaknesses

- Developing a prototype of intervention on such a small number of patient-caregiver dyads and proceeding to a pilot RCT seems premature especially given the reasons stated below.
- Grouping of all patients as “some form of dementia” without discerning different stages and (especially with such a small number) may result in a biased result. Patients who are in different stages of dementia have different needs and therefore different needs that caregivers may need in terms of additional resources.
- Consideration of how the “ADRD” or dementia diagnosis was derived for patient selection – chart history, report by informant, prior formal assessment, etc. would be important. Dementia diagnosis should have been established on outpatient basis when the patients were not acutely ill.
- Incorporating understanding of patient/caregiver factors influenced by gender, race/ethnicity, caregiver type (e.g., spouse/partner, children, friend, etc.), caregiver network (e.g., sole provider, extended family, etc.) in both the recruitment strategy and analysis would be important.
- Setting of the study and how this would influence control group – while an Acute Care for Elders (ACE) program will provide an ideal patient population, it is a fairly exclusive program very much geared towards providing enhanced care for older adults (i.e., not representative of the “real world”). When this is used as a setting, the control group and its outcome as a comparator may

not be ideal. The candidate addresses some of the limitations of conducting the study on the ACE Unit, and this is something that would need to be thought through.

4. Mentor(s), Co-Mentor(s), Consultant(s), Collaborator(s):
Strengths
- Mentors with very established track record of successful mentoring.

Weaknesses
- Dr. Farrar (primary mentor) – appears to have one manuscript in development with the candidate, but nothing published together thus far.

5. Environment:
Strengths
- Letter of commitment from Dr. Mason – Chair of Department of Kinesiology.
- Start-up funds to start the Geriatric Health Services Research Laboratory.

Weaknesses
- None noted.

Study Timeline:
Strengths
- Appropriate.

Weaknesses
- None noted.

Protections for Human Subjects:
Acceptable Risks and Adequate Protections.
- Appropriate.

Data and Safety Monitoring Plan:
Acceptable.
- Appropriate.

Inclusion Plans:
- Sex/Gender: Distribution justified scientifically.
- Race/Ethnicity: Distribution justified scientifically.
- Inclusion/Exclusion Based on Age: Distribution justified scientifically.

Training in the Responsible Conduct of Research:
Acceptable.
Format:
- Appropriate.

Subject Matter:
- Appropriate.

Faculty Participation:
- Appropriate.

Duration:
- Appropriate.

Frequency:
- Appropriate.

Budget and Period of Support:
Recommend as Requested.

THE FOLLOWING SECTIONS WERE PREPARED BY THE SCIENTIFIC REVIEW OFFICER TO SUMMARIZE THE OUTCOME OF DISCUSSIONS OF THE REVIEW COMMITTEE, OR REVIEWERS' WRITTEN CRITIQUES, ON THE FOLLOWING ISSUES:

PROTECTION OF HUMAN SUBJECTS: ACCEPTABLE. No concerns were reported by the scientific review group pertaining to the compliance to NIH regulations for the protection of human subjects.

INCLUSION OF WOMEN PLAN: ACCEPTABLE. No concerns were reported by the scientific review group regarding the enrollment of women.

INCLUSION OF MINORITIES PLAN: ACCEPTABLE. No concerns were reported by the scientific review group in relation to minority groups' recruitment. It was discussed that the participation of underrepresented groups (URG) is less than optimal. It is recommended that a more valiant effort be made to increase the inclusion of URG individuals to the research.

INCLUSION ACROSS THE LIFESPAN: ACCEPTABLE. No concerns were reported by the scientific review group regarding the inclusion throughout the lifespan; children exclusion is scientifically justified.

COMMITTEE BUDGET RECOMMENDATIONS: The budget was recommended as requested.

Footnotes for 1 K23 AG080068-01; PI Name: Fields, Beth

NIH has modified its policy regarding the receipt of resubmissions (amended applications).See Guide Notice NOT-OD-18-197 at https://grants.nih.gov/grants/guide/notice-files/NOT-OD-18-197.html. The impact/priority score is calculated after discussion of an application by averaging the overall scores (1-9) given by all voting reviewers on the committee and multiplying by 10. The criterion scores are submitted prior to the meeting by the individual reviewers assigned to an application, and are not discussed specifically at the review meeting or calculated into the overall impact score. Some applications also receive a percentile ranking. For details on the review process, see http://grants.nih.gov/grants/peer_review_process.htm#scoring.

MEETING ROSTER

Career Development for Clinicians/Health Professionals Study Section
National Institute on Aging Initial Review Group
NATIONAL INSTITUTE ON AGING
Career development for clinicians/health care professionals

AGCD-3
06/06/2022 - 06/07/2022

Notice of NIH Policy to All Applicants: Meeting rosters are provided for information purposes only. Applicant investigators and institutional officials must not communicate directly with study section members about an application before or after the review. Failure to observe this policy will create a serious breach of integrity in the peer review process, and may lead to actions outlined in NOT-OD-22-044 at https://grants.nih.gov/grants/guide/notice-files/NOT-OD-22-044.html, including removal of the application from immediate review.

CHAIRPERSON(S)

KELLEY, AMY STEVES, MD
PROFESSOR
BROOKDALE DEPARTMENT OF GERIATRICS AND PALLIATIVE MEDICINE
ICAHN SCHOOL OF MEDICINE AT MOUNT SINAI
NEW YORK, NY 10029

KIND, AMY J., MD, PHD
PROFESSOR & DIRECTOR
CENTER FOR HEALTH DISPARITIES RESEARCH
SCHOOL OF MEDICINE AND PUBLIC HEALTH
UNIVERSITY OF WISCONSIN
MADISON, WI 53705

MACK, WENDY JEAN, PHD
PROFESSOR
DEPARTMENT OF POPULATION AND PUBLIC HEALTH SCIENCE
UNIVERSITY OF SOUTHERN CALIFORNIA
LOS ANGELES, CA 90033

MEMBERS

ANTON, STEPHEN D, PHD, MS, BS *
ASSOCIATE PROFESSOR
DEPARTMENT OF AGING AND GERIATRICS
CHIEF
DIVISION OF CLINICAL RESEARCH
UNIVERSITY OF FLORIDA, COLLEGE OF MEDICINE
GAINESVILLE, FL 32611

BRICKMAN, ADAM M., PHD
PROFESSOR
TAUB INSTITUTE FOR RESEARCH ON ALZHEIMER'S DISEASE AND THE AGING BRAIN
DEPARTMENT OF NEUROLOGY
COLUMBIA UNIVERSITY
NEW YORK, NY 10027

CAMPBELL, NOLL L., PHMD, MS *
ASSISTANT PROFESSOR
DEPT. OF PHARMACY PRACTICE
PURDUE UNIVERSITY
INDIANAPOLIS, IN 46202

CHIN, JEANNIE, PHD *
ASSOCIATE PROFESSOR
DEPARTMENT OF NEUROSCIENCE
MEMORY AND BRAIN RESEARCH CENTER
BAYLOR COLLEGE OF MEDICINE
HOUSTON, TX 77030

KOROUKIAN, SIRAN M., PHD
PROFESSOR
DEPARTMENT OF POPULATION AND QUANTITATIVE HEALTH SCIENCES
CASE WESTERN RESERVE UNIVERSITY
CLEVELAND, OH 44106

KURELLA TAMURA, MANJULA, BS, MD, MPH *
PROFESSOR
DIVISION OF NEPHROLOGY
DEPARTMENT OF MEDICINE
STANFORD UNIVERSITY
STANFORD, CA 94305

LEW, SUSIE Q., MD *
PROFESSOR
DEPARTMENT OF MEDICINE
GEORGE WASHINGTON UNIVERSITY MEDICAL CENTER
WASHINGTON, DC 20037

MANNING, CAROL A, BA, PHD *
PROFESSOR
PROFESSOR AND DIRECTOR MEMORY DISORDERS CLINIC
DEPARTMENT OF NEUROLOGY
UNIVERSITY OF VIRGINIA
CHARLOTTESVILLE, VA 22908

MARSHALL, GAD ASHER, MD
ASSOCIATE PROFESSOR
DEPARTMENT OF NEUROLOGY AND
CENTER FOR ALZHEIMER RESEARCH & TREATMENT
BRIGHAM AND WOMEN'S HOSPITAL
BOSTON, MA 02115

MODY, LONA, MD *
HICKEY PROFESSOR OF INTERNAL MEDICINE
PROFESSOR OF EPIDEMIOLOGY
SCHOOL OF PUBLIC HEALTH
UNIVERSITY OF MICHIGAN MEDICAL SCHOOL
ANN ARBOR, MI 48105

MORENO, GERARDO, MD, MS
ASSOCIATE PROFESSOR
DEPARTMENT OF FAMILY MEDICINE
DAVID GEFFEN SCHOOL OF MEDICINE
UNIVERSITY OF CALIFORNIA, LOS ANGELES
LOS ANGELES, CA 90024

OH, ESTHER SEUNGHEE, MD, PHD *
ASSOCIATE PROFESSOR
DEPARTMENT OF PATHOLOGY
DIVISION OF NEUROPATHOLOGY
JOHNS HOPKINS UNIVERSITY SCHOOL OF MEDICINE
BALTIMORE , MD 21224

PROMISLOW, DANIEL EDWARD, PHD
PROFESSOR
DEPARTMENT OF LAB MEDICINE & PATHOLOGY AND
DEPARTMENT OF BIOLOGY
UNIVERSITY OF WASHINGTON
SEATTLE, WA 98195

WARNER, DAVID F., PHD, BS *
DEPARTMENT OF SOCIOLOGY
CASE WESTERN RESERVE UNIVERSITY
CLEVELAND, OH 44106

WOLK, DAVID A., MD
PROFESSOR
DEPARTMENT OF NEUROLOGY
UNIVERSITY OF PENNSYLVANIA
PHILADELPHIA, PA 19104

SCIENTIFIC REVIEW OFFICER

GRIMALDI, MAURIZIO, PHD, MD
SCIENTIFIC REVIEW OFFICER
NATIONAL INSTITUTE ON AGING
NATIONAL INSTITUTES OF HEALTH
BETHESDA, MD 20892

EXTRAMURAL SUPPORT ASSISTANT

NUCCI, JAMES BALDWIN
EXTRAMURAL SUPPORT ASSISTANT
SCIENTIFIC REVIEW BRANCH
NATIONAL INSTITUTE ON AGING
NATIONAL INSTITUTES OF HEALTH
BETHESDA, MD 20892

* Temporary Member. For grant applications, temporary members may participate in the entire meeting or may review only selected applications as needed.

Consultants are required to absent themselves from the room during the review of any application if their presence would constitute or appear to constitute a conflict of interest.
